# Photocatalytic degradation of paracetamol by semiconductor oxides under UV and sunlight illumination

**DOI:** 10.55730/1300-0527.3486

**Published:** 2022-07-19

**Authors:** Derman AKŞİT, Gülin Selda POZAN SOYLU

**Affiliations:** Department of Chemical Engineering, Engineering Faculty, İstanbul University-Cerrahpaşa, İstanbul, Turkey

**Keywords:** Photocatalytic degradation, paracetamol, semiconductor, UV-B and sunlight irradiation

## Abstract

In this study, photocatalytic degradation of paracetamol (acetaminophen), an analgesic and antipyretic drug, was investigated in using semiconductors under UV-B and sunlight irradiation. Semiconductor materials, Bi_2_O_3_, ZnO, TiO_2_, and ZrO_2_, were prepared using simple sol–gel, coprecipitation, and microwave synthesis methods. These photocatalyts were characterized by various spectroscopic and other techniques. As a result of the reactions, 25 ppm paracetamol with Bi_2_O_3_ and ZnO photocatalyst, total degradation was achieved with UV-B irradiation in 90 min. The results of photocatalytic activities showed that the highest degradation rate was obtained with the Bi_2_O_3_ photocatalyst. In the light of all findings, the nanorod-shaped Bi_2_O_3_ semiconductor which has a low band gap energy and high quantum efficiency exhibited significant effect on the activity despite of its low surface area.

## 1. Introduction

In recent years, many pharmaceutical drugs and hormones have been consumed in too much quantity. [1–3]. After the use of these drugs, negative effects on the environment and human health occur. Many unused, expired, and residual drugs are released into the environment during and after treatment. These pharmaceutical drugs are mixed with human urine and manure and wastewater after metabolism in the human body [4]. As a result of studies, pharmaceutical drugs have been detected in sewage, surface water, and groundwater in many countries [5–7].

Paracetamol (also known as acetaminophen or APAP) is an analgesic and antipyretic drug widely used all over the world [8,9]. It is also used as an intermediate in the production of azodies and photographic chemicals. The presence of trace amounts of paracetamol (PAR) and other xenobiotic compounds in drinking water is also a public health problem. Because these compounds are taken through drinking water for a long time, chronic health effects occur [10].

Some physicochemical and biodegradation methods are used to prevent contaminations and to reduce their concentration [11–13]. These methods have disadvantages such as being less effective and needing long reaction times.

It has been reported that some techniques such as activated carbon adsorption [14,15] ultrasound [16], Fenton oxidation [17,18] and ozonation [19,20] are frequently used in the removal of PAR in wastewater. However, it is noteworthy that there are few studies on the use of photocatalysts in the removal of pharmaceutical chemicals.

Advanced oxidation process (AOP) is an important technique known as photocatalytic oxidation of organic materials. After the photolysis reaction with UV light, the surface of the pollutants react with the catalyst and the electrons move from the valence band to the conduction band. Reactive species (e− and h+) initiate oxidation and reduction reactions. As a result of the redox reactions occurring with these active species, the mineralization of organic compounds takes place. Oxidation to strong oxidizing species such as OH• occurs by the reaction of light-formed cavities in the valence band with OH^−^ or H_2_O. Since the hydroxyl radical has an oxidation potential of 2.80 eV, it can react with organic compounds 1012 times faster than other oxidants [21].

Laboratory-scale studies and reports from treatment plants prove that paracetamol PAR shows very low or no biodegradability [22,23]. When an additional treatment such as chlorination is applied, very toxic by-products are formed [24,25]. Therefore, there is a need to investigate highly efficient, economical, and environmentally friendly processes such as AOPs.

It is important to develop harmless, highly effective alternative methods using nanocatalysts with low toxicity, low cost, and high performance, especially in the degradation of resistant hazardous chemicals.

Shakir et al. investigated the efficiencies of pure ZnO and lanthanum-doped ZnO in the photocatalytic degradation of paracetamol PAR. It was determined that the PAR solution was photocatalytically decomposed with ZnO and different lanthanum-doped ZnO under visible light, and the 3% lanthanum-doped ZnO nanoparticles achieved the highest photocatalytic activity. It was reported that approximately 80% of PAR decomposed in 180 min with 3% La-doped ZnO nanoparticles [26].

Moctezuma et al. investigated the oxidation of PAR under UV light with TiO _2_ catalyst in the presence of constant oxygen flow (100 mL/min). As a result, 100% conversion and 72% mineralization values were reached after 4 h [27].

In the literature, Bi_2_O_3_ is preferred as an alternative photocatalyst for the degradation of pollutants due to its lower band energy of 2.85 eV. Due to its superior properties permeability and distinctive photoluminescence properties, it is commonly used in gas sensors, fuel cells, optical coatings, ceramic glass production.

Sood et al. synthesized α-Bi_2_O_3_ and they explained that α-Bi_2_O_3_ nanorods catalyst exhibited excellent solar-light driven photocatalysis towards rhodamine B (97% dye degradation in 45 min) and 2,4,6-trichlorophenol (88% dye degradation in 180 min) [28].

Khairnar et al. prepared α-Bi_2_O_3_ by sol–gel method and investigated photocatalytic degradation of chlorpyrifos and methylene. The results showed that the synthesized α-Bi_2_O_3_ NPs shows the excellent photocatalytic efficiency against the MB dye as compared to the CPS pesticide under UV–visible irradiation [29].

Abu-Dief et al. synthesized monoclinic bismuth oxide nanorods (α-Bi_2_O_3_ NRs) by a simple one-step hydrothermal route. The as-synthesized α-Bi_2_O_3_ NRs photocatalyst exhibited better performance for degradation and decolorization of Methylene blue (MB) under ultraviolet (UV) irradiation. MB was completely photodegraded after 210 min under UV irradiation using α-Bi_2_O_3_ NRs as photocatalyst [30].

Bi_2_O_3_ is an effective photocatalyst for the degradation of organic compounds. In the literature, Bi_2_O_3_ has mostly been used for dyestuff degradation. However, the use of Bi_2_O_3_ in the photocatalytic degradation reactions of paracetamol has never been found. For this reason, the Bi_2_O_3_ catalyst was prepared by an easy and inexpensive method such as coprecipitation. The investigation of photocatalytic disruption efficiency in various reaction parameters may contribute to the research in the literature.

In our study for this purpose, the photocatalysts (TiO_2_, ZnO, ZrO_2_, and Bi_2_O_3_) to provide high efficiency in a short time were developed and the degradation of PAR, which is a widely used reagent in medicine, was investigated in the presence of these catalysts.

Photocatalysis is one of the most promising methods for water pollutant degradation with an unlimited energy source. The main aim is to investigate the effects of metal oxides with different band gap energies and different surface properties on the photodegradation of PAR. Another aim of the study is to provide total mineralization of PAR in the water phase in a short time or to reduce its concentration. In addition, the preparation of pure metal oxides using different synthesis methods and the elucidation of the structures of these catalysts constitute an important part of the study. The various characterization methods such as X-ray diffraction (XRD), scanning electron microscopy (SEM), diffuse reflectance spectroscopy (DRS), Brunauer, Fourier transform infrared (FTIR), and Emmet and Teller (BET) were used to determine the surface and optical properties of photocatalysts. The effects of the obtained results on the photocatalytic activity are explained.

## 2. Experiments and methods

### 2.1. Materials

In this experimental study, the basic materials were commercially available and used without further purification. These materials are bismuth (III) nitrate pentahydrate (98%; Alfa Aesar Company), zinc nitrate hexahydrate (ACROS Organics, 98%), titanium tetrachloride (Fluka, 99%), zirconium(IV) oxynitrate hydrate (Fluka). PAR, p-aminophenol, benzoquinone, and hydroquinone were purchased from Aldrich. Other chemicals, including nitric acid (65%), ethanol (absolute), acetonitrile (for HPLC, ≥99%), ammonia solution (25% in water), and sodium hydroxide (97%) were all purchased from Merck.

### 2.2. Catalyst synthesis methods

Bi_2_O_3_ was prepared with the coprecipitation method. After bismuth nitrate pentahydrate was dissolved in the nitric acid-water mixture, sodium hydroxide solution was added under stirring until the pH was 11. Heating was done until the temperature of the mixture reached 75 °C and kept at constant temperature for 2 h. The precipitate formed after waiting was filtered and washed with distilled water and absolute ethanol. Afterwards, the particles were dried at 80 **°C for 2** h and calcined by heating at 450 **°C for 2** h at 10 **°**C/min.

ZnO and ZrO_2_ were prepared with the coprecipitation method. The determined amounts of zinc nitrate hexahydrate and zirconium (IV) oxide nitrate hydrate were dissolved in deionized hot water and heated to 65 °C. The precipitation process was carried out by adding ammonia solution (25% by weight) drop by drop until the pH values of the solution reached 10. The mixture was then stirred at 65 °C for 2 h. Then the solutions were kept in a 500 W microwave oven for 3 min. The precipitate formed after waiting was filtered and washed with distilled water. Afterwards, the particles were dried at 100 **°C for 2** h and calcined by heating at 500 **°C for 5** h at 10 °C/min.

TiO_2_ structure was prepared with the simple sol–gel method. The determined amount of titanium tetrachloride was added dropwise into absolute ethanol under stirring. The formation of a transparent-yellow solution was observed. The resulting sol, after standing for a few days to form a gel, was dried in an oven at 105 °C for 24 h and, after grinding, calcined at 600 °C for 4 h.

### 2.3. Catalyst characterization

The surface area of the prepared metal oxides was measured by the nitrogen adsorption/desorption technique using the Quantachrome device. All catalysts were held under vacuum at 200 °C for 4 hours.

The crystal structure of the catalysts was determined with the Rigaku D/Max-2200 powder X-ray diffraction measuring device using CuKa radiation (λ= 1.54056 ε). The diffraction patterns were evaluated in the range of 10°–90° at a scan rate of 2 degrees 2θ. The crystallite size (D_avg_) values were figured out by the Debye-Scherrer equation by using FWHM values.

The morphologies and size distributions of nano-sized metal oxides were examined by high-resolution scanning electron microscopy (SEM) (JEOL/JSM-a6335F).

The presence of OH^−^ groups in powder samples were examined with FT-IR spectroscopy (Perkin Elmer Precisely Spectrum One).

The band gap energies of the semiconductor oxides were measured with the UV-Vis DRS technique and the absorption band gap energy (Eg) was calculated using the Kubelka-Munk function [F(R)] (Equation 1) [31].


Equation 1 $$F(R)=\frac{{\left(1+R\right)}^{2}}{2R}=\frac{\text{a}}{s}$$

Here n is the number, it determines the transition characteristic in the semiconductor and is equal to 2 for a direct allowed transition and ½ for an implicitly allowed transition. While making the calculations, n=1/2 was taken into account for the catalysts (Equation 2).


Equation 2 (αhυ)1/2=K(hυ-Ex)

K: A constant depending on the material
h: Planck’s constant (4.135 × 10–15 eV.s)
υ: Frequency of the photon

To determine the band gap energy (Eg) of the synthesized catalysts, a graph was drawn between the modified Kubelka–Munk function and the energy (E) of the absorbed light. Then, by drawing a tangent to the x-axis over the inflection point in the absorption band, the energy value at the point where this tangent cuts the x-axis was determined as the band gap energy of the catalyst.

### 2.4. Studies on photocatalytic activity

The heterogeneous reactions were carried out in a quartz two-neck vessel with a volume of 100 mL. After 0.1 g of catalyst was added to 50 mL of 25 ppm paracetamol solution, the adsorption-desorption equilibrium was reached by mixing for 30 min in the dark. Then, the light was turned on and samples were taken at certain time intervals. All photocatalytic reactions were carried out at pH 5. The photocatalytic reactions carried out using different light sources (UV-B (64 W), visible and natural sunlight) was investigated.

The paracetamol concentration was measured by HPLC analysis under certain conditions. Before analysis, samples were filtered through a PTFE fitler (45 **μm** diameter). During the analysis, a mixture of 35% acetonitrile - 65% water was used at a flow rate of 1 mL/min as the mobile phase.

## 3. Conclusion and discussion

### 3.1. Structural and optical properties of metal oxides

TiO_2_, ZnO, ZrO_2_, and Bi_2_O_3_ were measured as 40, 28, 96, and 12 m^2^ /g, respectively. The results are shown in Table.

According to the BET surface area results, it is seen that the surface area of Bi_2_O_3_ is lower than other oxides. However, photocatalytic activity is not only evaluated based on high BET surface area. It is known that the number and distribution of active sites in catalytic reactions is more important than the high of the surface area.

**Figure 1:**
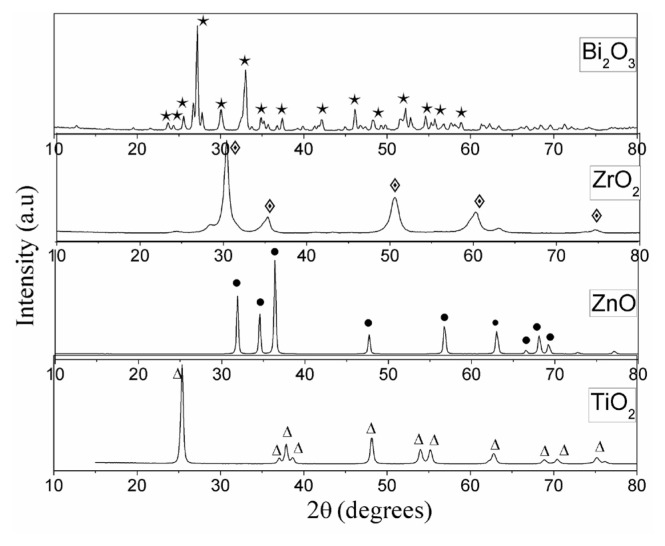
XRD patterns of catalysts.

The XRD diagrams of the catalysts are given in Figure 1. Anatase TiO_2_ phase (JCPDS card no. 89-4921), hexagonal phase ZnO (JCPDS 36-1451), tetragonal phase ZrO_2_ (JCPDS card no. 33-1483), and monoclinic phase α-Bi_2_O_3_ (JCPDS 41-1449) crystal structures were detected. The crystallite sizes of the catalysts were figured out according to the Scherrer formula and the outcomes are presented in Table.

**Figure 2:**
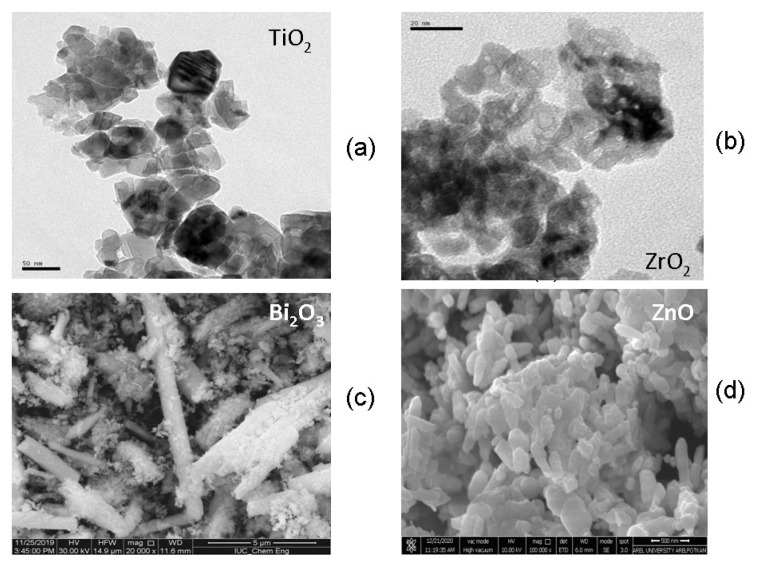
SEM images of catalysts

TEM and SEM analyses were performed to determine the morphology of the semiconductors, the particle distribution in the structure and the images are given in Figures 2a–2d. According to the TEM images, the particle size of pure TiO_2_ is irregular and the average particle size is around 65 nm, while the particle size of pure ZrO_2_ is 6 nm. From the SEM images, the clusters in cubic form were observed in the pure ZnO structure and the particle size of pure ZnO was around 80 nm. Bi_2_O_3_ rod-like structures of different lengths and thicknesses were observed in the SEM image.

FTIR-ATR analysis was performed to detect the hydroxyl group that supports degradation in photocatalytic reactions. During photocatalytic degradation, surface OH^−^ groups act not only to form hydroxyl radical (•OH) but also as active sites for adsorption of reactants [32]. As a result of the analysis, no hydroxyl groups were observed in the infrared spectrum of the photocatalysts. This result shows that most of the adsorbed water after heat treatment is removed from the catalyst surface [32].

Diffuse reflection spectroscopy (DRS) is used for the determination of optical properties of semiconductor materials and is effective in accurately estimating the optical band gap of the powder. The absorption band gap energy (Eg) can be determined with the Kubelka–Munk function [F(R)] [31].

To determine the band gap energy (Eg) of the synthesized catalysts, a graph is drawn between the modified Kubelka–Munk function and the energy (E) of the absorbed light. Then, a tangent to the x-axis is drawn over the inflection point in the absorption band and the energy value at the point where this tangent cuts the x-axis shows the band gap energy of the catalyst.

**Figure 3:**
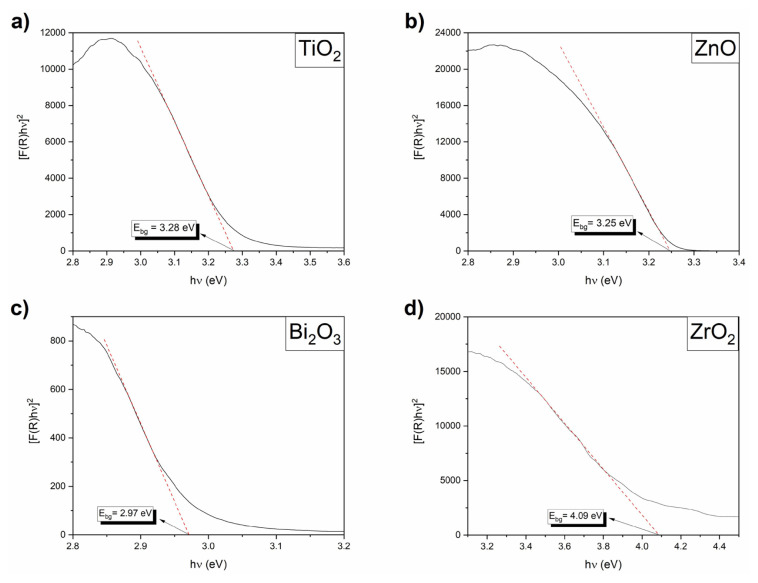
DRS spectra of TiO_2_, ZnO, Bi_2_O_3_, and ZrO_2_ catalysts.

The UV-vis diffuse reflection spectra of the catalysts are shown in Figure 3. The calculated band gap values of the catalysts are given in Table. The band gap of pure ZrO_2_, calculated highest band gap, is 4.09 is eV. The band gaps of TiO_2_ and ZnO are about 3.28 and 3.25 eV, respectively. As seen in Table, the lowest band gap energy (2.97 eV) belongs to the α-Bi_2_O_3_ catalyst.

PL analysis provides benefits in determining the efficiency of the load carrier capture, migration, and transfer. In addition, semiconductors of PL spectra are also used to derive the band interval determination and recombination mechanism. As a result of the recombination of stimulated electrons and holes, changes occur in the number of electron-holes in semiconductor particles [33]. A low PL density means the low recombination rate of the electron hole under the light radiation [34].

**Figure 4 f4-turkjchem-46-6-1866:**
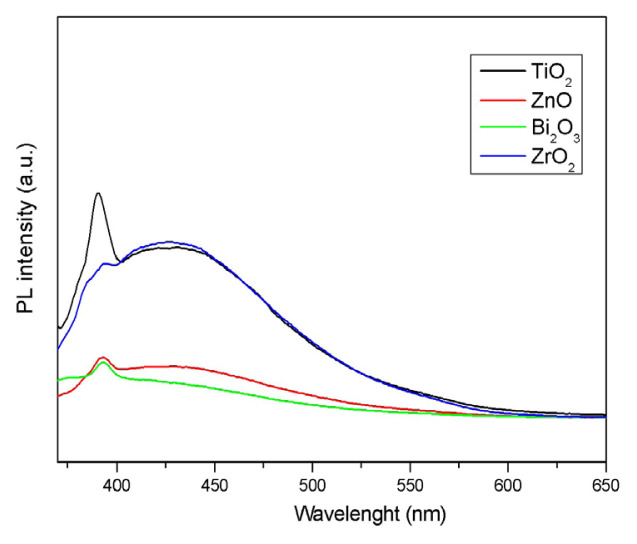
PL spectra of Bi_2_O_3_, ZnO, ZrO_2_ and TiO_2_ catalysts.

The PL spectra of the photocatalysts are given in Figure 4. The PL emission spectra of the samples were found to show major peaks at similar positions at different intensities. It is understood that the PL intensity of the Bi_2_O_3_ emission spectrum is the lowest and the electrons and holes show a low recombination rate. It can be concluded that the use of the Bi_2_O_3_ catalyst improves the photoactivity as the electrons and holes reduce recombination.

### 3.3. Photocatalytic activity results

It is known that the photocatalytic degradation reaction of paracetamol occurs in a pseudo-first-order reaction [26].

The Langmuir–Hinshelwood (LH) kinetic model was applied for the kinetic evaluation of the reactions [35]. The kinetic model is shown in Equation 3.


Equation 3 $$\text{ln}({\text{C}}_{\text{o}}/{\text{C}}_{\text{t}})={\text{k}}_{\text{obs}}\;\text{t}$$

Here, in the reaction with low initial PAR concentration, Co is the initial PAR concentration, C_t_ t is the PAR concentration at time, and k_obs_ (min^−1^) is the rate constant of the observed pseudo-first order reaction. To compare the activities of the catalysts, the photocatalytic degradation efficiencies and reaction rates were calculated and the activity values are given in Table.

In addition, after the experiments, the photocatalytic degradation efficiencies were figured out and the values are given in Table.

The decomposition performance of the PAR (%R) is calculated with Equation 4:


Equation 4 $$\%\text{R}=\frac{C_{o}-C}{C_{o}}\times 100$$

Photocatalytic degradation reactions of PAR were carried out at pH 5 in the presence of Bi_2_O_3_, ZnO, ZrO_2_, and TiO_2_ catalysts. The PAR solution was kept in the dark for 30 min under stirring to ensure adsorption-desorption equilibrium. Then, the light was turned on and samples were taken at certain time intervals, and the concentration changes were followed. The variation of PAR concentration with time was followed by HPLC analysis.

**Figure 5 f5-turkjchem-46-6-1866:**
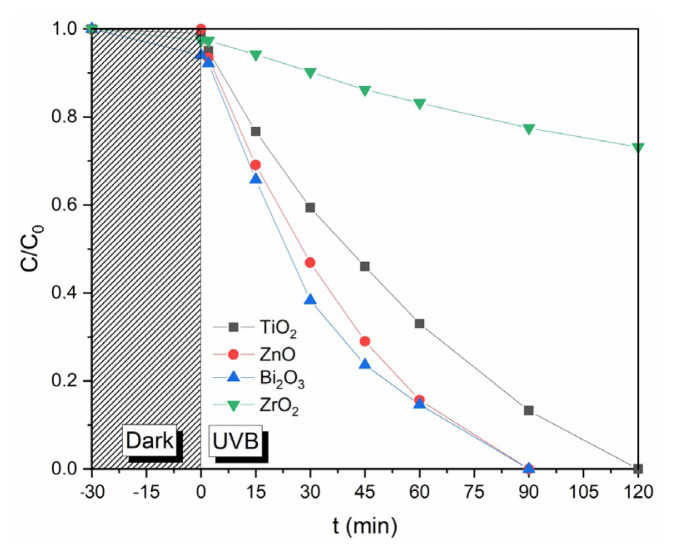
The photocatalytic degradation of PAR in Bi_2_O_3_, ZnO, ZrO_2_, and TiO_2_ catalysts with UV-B irradiation.

Figure 5 indicates the concentration differences in the reaction with various metal oxides under UV-B illumination. In the calculated PAR removal after 90 min, the photocatalytic efficiencies are determined in decreasing order as follows: Bi_2_O_3_ (100%) > ZnO (100%) > TiO_2_ (86.76%) > ZrO_2_ (22.53%). In the presence of Bi_2_O_3_ and ZnO catalysts, it was observed that PAR was completely degraded within 90 min. Bi_2_O_3_ catalyst provided a higher degradation rate than the ZnO catalyst. However, ZnO nanoparticles showed very low photocatalytic activity under natural sunlight. The band gap energy of ZnO nanoparticles is greater than that of Bi_2_O_3_. It can be said that the band gap energy of ZnO nanoparticles is higher than that of Bi_2_O_3_.

When the optical reflectance spectra of the photocatalysts are examined, it is understood that the wavelengths are between 200 and 500 nm, which indicates the presence of visible light adsorption in addition to a strong UV light region. Among these semiconductors, only the Bi_2_O_3_ catalyst tends to shift towards the visible region. Due to this feature, Bi_2_O_3_ catalyst showed activity in both UV and visible regions.

**Figure 6 f6-turkjchem-46-6-1866:**
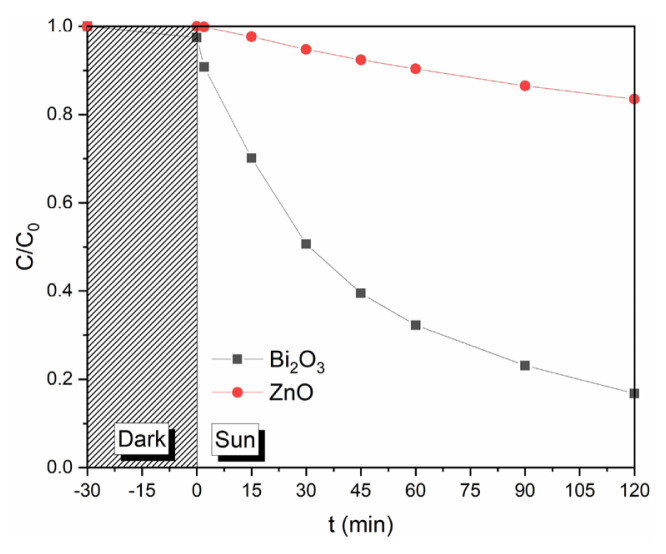
The photocatalytic degradation of PAR on Bi_2_O_3_ and ZnO catalysts with natural sun irradiation.

The photodegradation of PAR under sunlight with Bi_2_O_3_ and ZnO nanostructures are shown in Figure 6. According to the results, PAR degradation efficiencies of 76.89% and 21.63% were obtained over Bi_2_O_3_ and ZnO within 90 min of the reaction, respectively.

In addition, the reusability of the Bi_2_O_3_ catalyst was studied on fresh dye samples (5 trials). Bi_2_O_3_, when used for the first time, could degrade 100% PAR, with a small change (to 96.82%) in the efficiency when used for five times. This decrease in the efficiency for Bi_2_O_3_ catalyst resulted probably from the photocorrosion effect.

**Figure 7 f7-turkjchem-46-6-1866:**
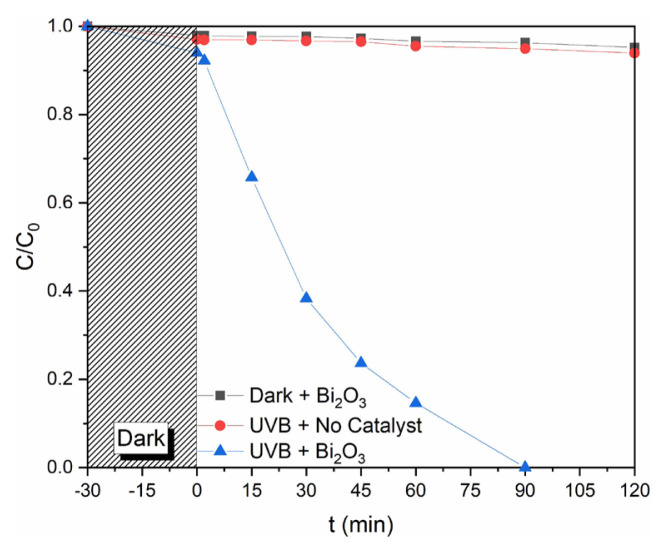
The comparison of photolysis, adsorption, and photocatalysis studies of PAR with Bi_2_O_3_.

Moreover, we also studied in various processes to determine the effects of adsorption (without the light exposure) and photolysis under UV-B light (no catalyst) and the degradation of PAR on the photocatalytic activity of Bi_2_O_3_ and the results of these comparative studies are showed in Figure 7. The degradation results indicate the negligible change for photolysis under UV-B and insignificant adsorption of PAR onto Bi_2_O_3_ within 120 min of reactions. This also suggests that stereochemical configuration of PAR is unsuitable to chelate with Bi, leading to negligible chemical adsorption of PAR onto Bi_2_O_3_ surface. However, in the presence of Bi_2_O_3_ with UV-B radiation, much faster degradation of PAR occurred compared to reactions without Bi_2_O_3_ and radiation only.[Fig f4-turkjchem-46-6-1866][Fig f5-turkjchem-46-6-1866][Fig f6-turkjchem-46-6-1866][Fig f7-turkjchem-46-6-1866]

Wanget al. prepared Pd-BiVO_4_ catalyst with the impregnation method. They reported that Pd-BiVO_4_ achieved 100% removal and reached up to 40% TOC removal in 1 h under visible light irradiation [36]. In another study, photodegradation of paracetamol at a wavelength of radiation of 254 nm with TiO_2_ nanotubes was studied with UV-spectroscopy, HPLC and measurement of the potential zeta in dependence of the solution pH. The efficiency of the photodegradation of paracetamol PAR (20 mg L^−1^) was 99% after 100 min of UV-B light exposure [37].

Finally, the outcomes of these studies provide a concise viewpoint in this important research area and specifically propose further research opportunities in the photocatalytic performance of the other metal oxide nanoparticles and widening the scope of their potential photocatalytic applications.

## 4. Conclusion

The semiconductor materials were prepared with the coprecipitation and sol–gel methods. The structural properties of these materials were characterized by various techniques. After that, the effect of semiconductor oxides with different crystal sizes and band gaps on the photocatalytic degradation of paracetamol was investigated. Additionally, the efficiencies of photocatalysts in degradation reactions performed under UV-B light and natural sunlight were compared. As a result of photocatalytic reactions, the highest degradation rate was obtained onto Bi_2_O_3_ under UV-B light. Moreover, nanorod -structured Bi_2_O_3_ and cubic-structured ZnO photocatalysts was achieved in 90 min under UV-B light.

## Figures and Tables

**Table:** The crystallite size, specific surface area, band gap, morphology of materials, reaction rate constant, and paracetamol (PAR) degradation efficiency over 90 min (%) under UV-B irradiation.

Catalysts	Crystallite Size (nm)	S_BET_ (m^2^g^−1^)	Band gap (eV)	Morphology	Paracetamol (PAR) degradation efficiencies (%)	k_r_ (min^−1^ )	R^2^
**α-Bi**_2_O_3_	41	12	2.97	Monoclinic	100	0.032	0.998
TiO_2_	43	40	3.28	Anatase	86.76	0.018	0.999
ZnO	64	29	3.25	Hexagonal	100	0.029	0.989
ZrO_2_	7	95	4.09	Tetragonal	22.53	0.003	0.998
